# Temporally and Spatially Constrained ICA of fMRI Data Analysis

**DOI:** 10.1371/journal.pone.0094211

**Published:** 2014-04-11

**Authors:** Zhi Wang, Maogeng Xia, Zhen Jin, Li Yao, Zhiying Long

**Affiliations:** 1 State Key Laboratory of Cognitive Neuroscience and Learning & IDG/McGovern Institute for Brain Research, Beijing Normal University, Beijing, China; 2 Center for Collaboration and Innovation in Brain and Learning Sciences, Beijing Normal University, Beijing, China; 3 School of Information Science and Technology, Beijing Normal University, Beijing, China; 4 Laboratory of Magnetic Resonance Imaging, Beijing 306 Hospital, Beijing, China; Vanderbilt University, United States of America

## Abstract

Constrained independent component analysis (CICA) is capable of eliminating the order ambiguity that is found in the standard ICA and extracting the desired independent components by incorporating prior information into the ICA contrast function. However, the current CICA method produces constraints that are based on only one type of prior information (temporal/spatial), which may increase the dependency of CICA on the accuracy of the prior information. To improve the robustness of CICA and to reduce the impact of the accuracy of prior information on CICA, we proposed a temporally and spatially constrained ICA (TSCICA) method that incorporated two types of prior information, both temporal and spatial, as constraints in the ICA. The proposed approach was tested using simulated fMRI data and was applied to a real fMRI experiment using 13 subjects who performed a movement task. Additionally, the performance of TSCICA was compared with the ICA method, the temporally CICA (TCICA) method and the spatially CICA (SCICA) method. The results from the simulation and from the real fMRI data demonstrated that TSCICA outperformed TCICA, SCICA and ICA in terms of robustness to noise. Moreover, the TSCICA method displayed better robustness to prior temporal/spatial information than the TCICA/SCICA method.

## Introduction

Independent component analysis (ICA) is a data-driven method that can recover a set of maximally independent sources from observed multivariate data without using any prior information [1], [2], [3]. Functional magnetic resonance imaging (fMRI) is a widely used noninvasive neuroimaging technique that measures hemodynamic responses to reveal the functions of the brain. ICA has gained increasing acceptance in the functional imaging research community [4], [5], [6] since McKeown [7] first proposed the application of spatial ICA in fMRI data analysis. In contrast to the complementary univariate general linear model (GLM) method, which is performed on a voxel-by-voxel basis [8], the ICA method is able to extract multiple brain networks that are engaged in various elements of cognitive processing without any prior knowledge. This ability makes ICA an increasingly attractive exploratory tool to study functional brain networks either at rest [9] or during a cognitive task [10].

Although ICA is not dependent on any prior information, some previous studies have suggested that the performance of ICA can be improved by incorporating prior temporal information [3], [11]. Of note, both the variances and the order of the independent components (ICs) that are estimated by the standard ICA are arbitrary. The order indetermination in ICA leads to the problem of target component selection. Additional prior information can contribute to the solution to this problem, even if the prior information was incomplete. Luo et al. (1999) first proposed a principal independent component analysis concept that could extract objective independent components directly by introducing some asymmetric information to the network [12]. The constrained independent component analysis (CICA) that was proposed by Lu and Rajapakse (2000, 2005) could automatically extract the desired components in a predefined order and reduce the computational cost by introducing constraints into the classical ICA [13], [14]. CICA considers only one type of prior information to produce constraints that can act on either the source matrix [14] or the mixing matrix [15]. For fMRI data analysis, CICA was adopted to extract temporally independent components that were related to the task by using temporal constraints on the source matrix without separating all of the sources [14]. Lin (2010) applied CICA to estimate the desired spatially independent components from fMRI data using spatial constraints on the sources matrix [16]. To improve the convergence of CICA, learning-rate-free CICA algorithms were proposed by Wang (2011) and were applied to separate spatially independent components from fMRI data using temporal constraints on the mixing matrix [17]. Rasheed (2009) proposed the constrained spatiotemporal ICA that used two cascaded CICA stages, one for each domain, to determine the maximally independent yet desired sources in both the spatial and temporal domains [18]. A priori information that was available in the spatial/temporal domain was fed to the first CICA stage, and the output of the first CICA stage was added to the second CICA stage as the constraint.

In spite of the available temporal and spatial prior information in the fMRI data, these previous studies on CICA only included one type of prior information (temporal/spatial) as constraints, which can increase the dependency of CICA on the accuracy of the prior information. It is impossible in most applications to obtain accurate prior temporal or spatial information in fMRI data before ICA processing. This situation is particularly the case for the prior spatial information of fMRI data. Moreover, how the accuracy of this prior information affects the performance of CICA remains unknown. Accordingly, it is important to investigate how to reduce the dependency of CICA on the accuracy of prior information.

Based on the above considerations, this study aimed at exploring a method to reduce the impact of the accuracy of prior information on CICA and to improve CICA performance. The temporally and spatially constrained ICA (TSCICA) method was proposed by introducing dual constraints into ICA within the framework of CICA. Although spatially/temporally independent components can be identified using a spatial/temporal ICA from fMRI data, the spatial ICA is much more widely used than the temporal ICA [19]. Therefore, the spatial ICA was used for TSCICA in the current study, although the temporal ICA is also suitable for use in TSCICA. The basic idea of TSCICA was to simultaneously incorporate the temporal constraints on the mixing matrix and the spatial constraints on the source matrix into the spatial ICA. Using simulated and real fMRI experiments, we compared TSCICA with GLM, ICA, the temporally CICA (TCICA) method, which introduced temporal constraints on the mixing matrix, and the spatially CICA (SCICA) method, which introduced spatial constraints on the source matrix. The results from both the simulated and real fMRI experiments demonstrated that TSCICA outperformed TCICA, SCICA and ICA.

## Theory

### Constrained ICA

The spatial ICA model that is typically applied to fMRI data can be expressed as:
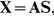
(1)where **X** is the K×V observed fMRI signal data. K is the number of scans, whereas V is the number of voxels. **A** is the K×C matrix and **S** is the C×V source matrix, where C is the number of total independent components. Each row of matrix **S** represents one of the spatially independent components, and each column of matrix **A** represents the time course of the corresponding independent component. The standard ICA algorithm aims to find a C×K unmixing matrix **W** such that the output **Y** = 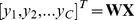 provides estimates of all of the source signals. Before ICA processing, the data are whitened by a standard principal component analysis (PCA) as follows:
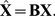
(2)The M×K matrix **B** in Eq. (2) is called the whitening matrix and can be easily determined through the following formula:
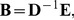
(3)where **D** is the M×M diagonal matrix of the M largest eigenvalues of the covariance matrix of the input data E{**XX** ^T^}a, and **E** is the M×K transposed matrix of the M eigenvectors that correspond to the M eigenvalues in matrix **D**. Thus, the whitened data 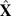 becomes M×V. Both FastICA and Informax ICA algorithms have been widely used in fMRI data analyses [20], [21], [22].

CICA is able to extract the desired source signals only by incorporating prior information into the contrast function in the form of inequality constraints and equality constraints. The CICA method is modeled as the following constrained optimization problem [13], [14]:
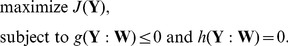
(4)


Similar to the FastICA algorithm, the contrast function *J*(**Y**) of CICA is set as negentropy. In general, we can obtain the following approximation:
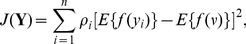
(5)The components of output **Y** = 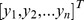 are mutually independent and correspond to *n* (<C) sources mixed in the observations, ρ*_i_* is a positive constant, and ν is a Gaussian variable with zero mean and unit variance. *f*(·) is a non-quadratic function, such that
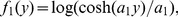
(6)

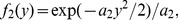
(7)

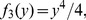
(8)where 1≤
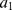
≤2 and 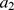
≈1.

*g*(**Y**: **W**) = [*g*_1_(*y*_1_: *w*_1_), *g*_2_(*y*_2_: *w*_2_), *g*_p_(*y*_p_: *w*_p_)]^T^ includes p inequality constraints, and *h*(**Y**: **W**) = [*h*_1_(*y*_1_: *w*_1_), *h*_2_(*y*_2_: *w*_2_), *h*_q_(*y*_q_: *w*_q_)]^T^ includes q equality constraints. The constraints *g*(**Y**: **W**) and *h*(**Y**: **W**) can act on either the mixing matrix or the source matrix. The CICA algorithm uses p inequality constraints *g*(**Y**: **W**) and q equality constraints *h*(**Y**: **W**) of either the temporal or spatial information to constrain *J*(**Y**) and uses Lagrange multipliers [23] to search for the optimal solution.

### Temporally and spatially constrained ICA

The proposed TSCICA method was provided within the framework of CICA. Suppose we want to extract n (n≥1) sources of interest from C (C>n) total source signals. The spatial reference signals 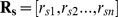 and the temporal reference signals 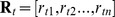 can be constructed from the prior spatial and temporal information of the n sources of interest. For an ideal separation **AW = I** and assuming that 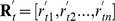 is the transformation of **R** *_t_* into the unmixing space, **R** *_t_'* can be expressed as:
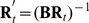
(9)


The proposed TSCICA method can be formulated in the framework of CICA.
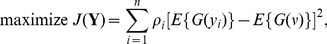
(10)

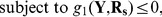
(11)

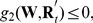
(12)

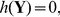
(13)where 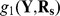 = [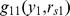,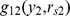,…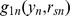]^T^ includes *n* inequality spatial constraints, and 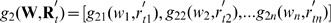 includes *n* inequality temporal constraints. 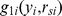 and 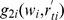 can be expressed as 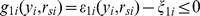 (*i* = 1,…,*n*) and 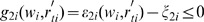, where 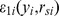 is the closeness measure between the output *y_i_* and the spatial reference *r_si_*, and 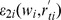 is the closeness measure between the output *w_i_* and the temporal reference 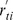. 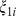
(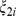) is a threshold that distinguishes the desired output *y_i_*(*w_i_*) from the other outputs. Moreover, the equality constraint 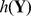 is added to ensure that the contrast function 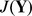 and the weight vector *w* were bounded, e.g., 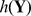 = [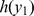,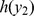,…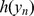]^T^ and 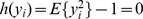 [13], [14]. Compared with the model (4) of CICA, TSCICA added two types of inequality constraints including 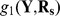 and 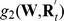 into the model. Based on the constraints of the equation (11)–(13), TSCICA can estimate the optimal solution of equation (10) using Lagrange multipliers. The corresponding augmented Lagrange function L of TSCICA is given by:
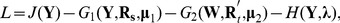
(14)where
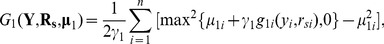
(15)

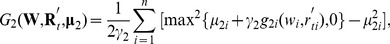
(16)

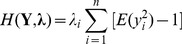
(17)

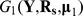
 and 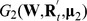 transform the original inequality constraints of the spatial and temporal reference signals into equality constraints; and **μ_1_** = [μ_11_, μ_12_,…, μ_1n_], **μ_2_** = [μ_21_, μ_22_, …, μ_2n_], and **λ_1_** = [λ_11_, λ_12_,…λ_1n_] are the vectors of positive Lagrange multipliers that correspond to the spatial inequality, temporal inequality, and equality constraints. γ_1_ and γ_2_ are the penalty parameters.

A Newton-like gradient method can be used to solve this optimization problem [13]:
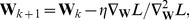
(18)where 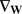 and 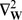 indicate the first and second derivatives, respectively, η is the learning rate, and *k* is the iterative step. The gradient of 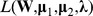 is given by:
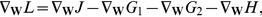
(19)where the matrix 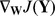 = [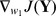,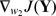,… 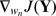]^T^ denotes the gradient of 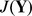 with elements 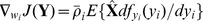. 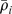 is the constant term and can be expressed as 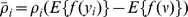. The term 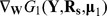 = [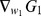, 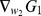, …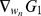]^T^ denotes the gradient of 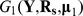 with element 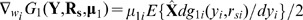, and 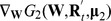 = [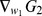, 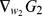,…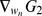]^T^ denotes the gradient of 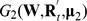 with element 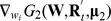 = 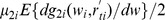. The last term is 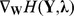 = [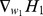,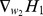,…,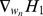] with elements 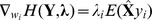. The 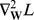 can be obtained using the following equation:
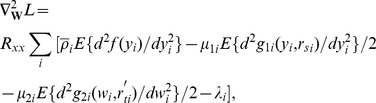
(20)where R_xx_ = E{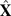 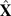 ^T^}. The optimum multipliers ***μ*** **_1_**, ***μ*** **_2_** and **λ** are iteratively updated based on following equation:
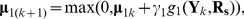
(21)

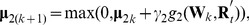
(22)

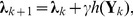
(23)where **W** can be initialized as a random matrix. At each iteration step, *w_i_* can be centered and normalized using the following equation to simplify the calculation and to improve the stability of the algorithm:
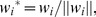
(24)where 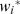 denotes the new value of *w_i_*. Additionally, the weight vectors are decorrelated at each step using the following equation to prevent different components from converging to the same solution [21], [22]:
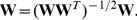
(25)The procedure of TSCICA is listed as follows:

Center and whiten the observed signal **X**;

Initialize ***γ*** **_1_**, ***γ*** **_2_**, ***ξ*** **_1_**, ***ξ*** **_2_** and ***λ***;

Initialize **W** as a random matrix. Center and normalize each row vector of **W**;

For the *k*th iterative step:

Update **W** using equations (18), (19) and (20).

Center and normalize *w_i_* using Eq. (24), and decorrelate the **W** matrix using Eq. (25).

Update ***ξ*** **_1_**, ***ξ*** **_2_**, ***λ*** using Eqs. (21), (22) and (23).

Repeat the above steps until either *g*_1_(**Y**) or *g*_2_(**W**)<0 and ||Δ**W**||<10^−4^.

When the iteration stops, the interested ICs can be obtained according to **Y** = **WX**.

## Materials and Methods

### 1. Ethics statement

The human fMRI experiment conducted in this study was approved by the Institutional Review Board of Beijing Normal University (BNU) Imaging Center for Brain Research, National Key Laboratory of Cognitive Neuroscience. All of the subjects gave written informed consent according to the guidelines set by the MRI Center of Beijing Normal University.

### 2. Simulation

The simulated fMRI data were generated to illustrate the robustness and the feasibility of the proposed TSCICA method. To investigate the impact of the accuracy of prior information on the ICA's estimation, the performance of TSCICA was compared with TCICA and SCICA under conditions of different prior temporal and spatial information.

The dimension of each simulated dataset were reduced by PCA, with 99.9% of the total variance of the mixed signals retained before TSCICA, TCICA, SCICA and ICA, to ensure that all the informative components were included [16]. The nonlinear function *f*(·) used equation (6), and the constant ρ in equation (10) was set to 1 [17]. For the TSCICA, TCICA and SCICA methods, the learning rate η was set to 0.98^k^, where k is the iteration count. Generally, the learning rate η was set as a fixed value. To reduce the computational cost and to ensure a stable convergence, we set the learning rate as an alterable value that reduced with an increasing iterative step. Based on the parameter setting in the previous study [17], the spatial penalty parameter *γ*_1_ was set to 0.1×4^(k−1)^, and the Lagrangian multipliers *μ*_1_ and *λ* were initialized to 1 in this study. Moreover, the Lagrangian multiplier *μ*_2_ was also initialized to 1 and the temporal penalty parameter *γ*_2_ was set to 0.2×4^(k−1)^. The use of this value for *γ*_2_ was validated in the following simulation. The correlation was used such that *ε*(*y_i_*, *r_si_*) = -E{*y_i_r_si_*} and *ε*(*w_i_*,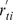) = -E{*w_i_* 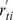}. The threshold *ξ*_1_ (*ξ*_2_) was initialized to 0.9 and was adjusted according to the correlation coefficient of the estimated *y_i_*(*w_i_*) and *r_si_* (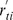) during each iteration. The spatial penalty parameter and the Lagrangian multiplier of SCICA were the same as *μ*_1_ and *γ*_1_ of TSCICA. The temporal penalty parameter and the Lagrangian multiplier of TCICA were the same as *μ*_2_ and *γ*_2_ of TSCICA. The termination criteria were set to ||Δ*w*||<10^−4^ for FastICA, ||Δ*w*||<10^−4^ combined with *g*_1_(*y*)<0 for SCICA, and ||Δ*w*||<10^−4^ combined with *g*_2_(*w*)<0 for TCICA. Moreover, the termination criteria for TSCICA were set to be either *g*_1_(*y*) or *g*_2_(*w*)<0 and ||Δ*w*||<10^−4^. A maximum of 200 iterations was allowed for each ICA decomposition run of each method. When an algorithm did not meet the convergence criteria as described, a new decomposition run was started. The target IC was transformed into a Z-score. The core FastICA algorithm was downloaded from the internet (http://www.cis.hut.fi/projects/ica/fastica/). TSCICA, TCICA and SCICA were developed using the programming language Matlab (Mathworks, Natick, MA, USA), which was based on the FastICA algorithm. The matlab codes of TSCICA algorithm can be downloaded from the website (http://cist.bnu.edu.cn:8080/infolab/files/TSCICA.rar).

The receiver operation characteristic (ROC) analysis [24] was applied to compare the spatial detection power of the different methods. The relation between the false positive ratio (FPR) and the true positive ratio (TPR) can be drawn as a ROC curve. The area under the curve can be used to evaluate the accuracy of the method. A larger area under the curve indicates that the method is more accurate.

#### Generation of temporal and spatial references

**Figure 1:**
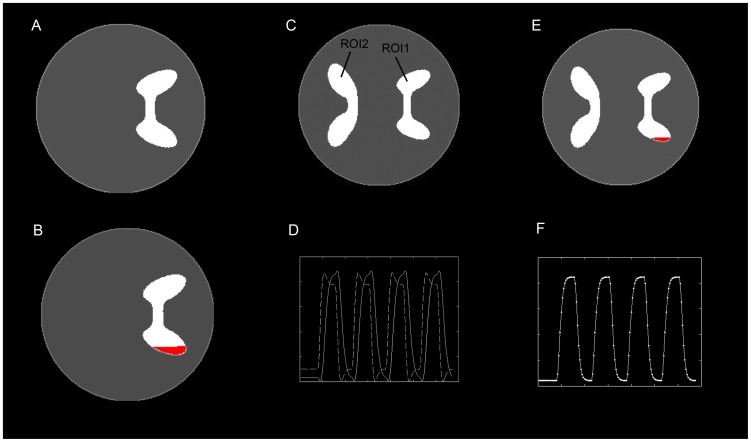
Pre-defined regions and the simulated fMRI response. (A) The predefined ROI for the simulated datasets including one task-related component. (B) One spatial reference with a 10% overlap rate applied to the simulated datasets including one task-related component. (C) The predefined ROI1 and ROI2 of the simulated datasets including two task-related components. (D) The simulated fMRI response added to the two ROIs. Solid line corresponds to the time course added to ROI1 and dotted line corresponds to the time course that was added to ROI2. (E) The spatial reference that was applied to the simulated datasets, including two task-related components. (F) The temporal reference that was applied to the simulated datasets, including two task-related components.

To investigate the robustness of the proposed method to the accuracy of the temporal reference, a set of temporal references, which were composed of 10 time courses that were derived from the convolution of the experimental paradigm with the hemodynamic response function (HRF), were generated. The HRFs that underlie the ten temporal references were determined by seven parameters of the spm_hrf function in the software SPM8 (Statistical Parametric Mapping, http://www.fil.ion.ucl.ac.uk/spm/software/). The seven parameters of the HRF varied across the ten temporal references. The temporal accuracy of each reference was measured using the correlation coefficient (CC) between the temporal reference and the true fMRI response that was added to the ROI. The correlation coefficients of the 10 temporal references varied from 0.5 to 0.95, with an increment of 0.05. Moreover, two sets of spatial templates were generated as different spatial references. The activated/nonactivated voxels in the templates were defined as one/zeros. It was assumed that N represents the number of voxels in the ROI in Fig. 1A, X represents the number of voxels that were in both the activated regions in the spatial reference and the ROI, and Y represents the number of voxels that were in the activated regions in the spatial references but not in the ROI. We defined the spatial overlap rate as X/N and the error rate as Y/N. The first set of spatial references was generated to investigate the robustness of the proposed method to the noise magnitude/spatial overlap rate and to determine the penalty parameter. This set of references were composed of 10 spatial templates that had a zero error rate and different overlap rates that varied from 1% to 10%, with an increment of 1%. Fig. 1B displays a spatial reference that contained the 10% overlap rate. The second sets of spatial references were generated to investigate the robustness of the proposed method to the spatial error rate. This set was composed of 100 spatial templates that had different overlap rates that varied from 1% to 10%, with an increment of 1%, and different error rates that varied from 1% to 10%, with an increment of 1%. Here, we called the temporal reference set TRef, with the first and second sets of spatial references called SRef1 and SRef2, respectively.

#### Robustness to the noise magnitude

Simulated datasets with different contrast-to-noise ratio (CNR) were generated using the SimTB toolbox (http://mialab.mrn.org/software/simtb/index.html) [25]. Each dataset contained 200×200 voxels. It was assumed that the simulated experiment included one task and that the task induced one task-related spatial component. The entire session lasted 270-s and consisted of four 30-s task blocks that alternated with five 30-s rest blocks. The region of interest (ROI) was predefined in the SimTB toolbox (see Fig. 1A). The simulated fMRI response that was added to the ROI was derived from a convolution of the stimulus paradigm using the HRF. The HRF was created by the spm_HRF function using the default parameters. Rician noise was added to each dataset relative to a specified CNR [26]. A CNR was defined as 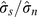, where 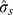 is the temporal standard deviation of the true signal and 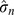 is the temporal standard deviation of the noise [25]. The CNR of the simulated datasets varied from 0.01 to 0.21, with an increment of 0.02. For each CNR level, ten simulated datasets were generated. Thus, 110 simulated datasets were produced in this experiment.

Two temporal references with high accuracy (CC = 0.8) and low accuracy (CC = 0.6) from TRef and two spatial references with high spatial overlap rate (8%) and low overlap rate (3%) from SRef1 were considered. TSCICA/SCICA/TCICA was applied to each dataset 4 times to automatically extract the desired task-related component. For each application, one spatial reference with a high/low spatial overlap rate was used as a spatial constraint of TSCICA and SCICA, and one temporal reference with high/low accuracy was used as a temporal constraint of TSCICA and TCICA. For each dataset, SCICA was performed 2 times using the same spatial reference, and TCICA was performed 2 times using the same temporal reference. The ROC area was recorded after each application. Additionally, each dataset was processed by FastICA 4 times. To identify the task-related component that was separated by FastICA, the temporal correlation between the time course of each component and one temporal reference with high/low accuracy was calculated. The component with the highest temporal correlation coefficient was selected. Thus, the temporal reference with high/low accuracy was used 2 times to identify the task-related component of each dataset for the 4 FastICA applications. Meanwhile, the ROC area was obtained for each TSCICA/SCICA/TCICA/FastICA application. To examine the impact of the noise level on TSCICA, SCICA, TCICA and FastICA, the mean values were calculated for 40 ROC areas at each noise level. The 40 ROC areas of TSCICA, SCICA and TCICA were obtained from 10 datasets ×4 TSCICA/SCICA/TCICA applications for each CNR. The nonparametric Wilcoxon [27] tests for paired samples were performed to further compare the difference in the ROC area between TSCICA and SCICA/TCICA/FastICA at each noise level.

Moreover, the target component that was extracted from one simulated dataset with a CNR equal to 0.03 by each ICA method was transformed into Z score. A spatial reference with an overlap rate that was equal to 2% was used in TSCICA and SCICA, and a temporal reference with a correlation coefficient that was equal to 0.9 was used in TSCICA and TCICA. To map the spatial activation of the target components, the voxels with a Z score that was higher than 2 were considered activated [7].

#### Robustness to spatial overlap rate, temporal accuracy and spatial error rate

In this experiment, 11 simulated datasets with different CNRs were generated using the identical method as the above experiment. The CNR of the 11 simulated datasets varied from 0.01 to 0.21, with an increment of 0.02.

To investigate the effect of the spatial overlap rate/temporal accuracy on TSCICA, TCICA and SCICA, each method was applied to each dataset 100 times to automatically extract the desired task-related component. For each application, one spatial reference from SRef1 was used as a spatial constraint of TSCICA/SCICA, and one temporal reference from TRef was used as a temporal constraint of TSCICA/TCICA. For each dataset, SCICA was performed 10 times using the same spatial reference, and TCICA was performed 10 times using the same temporal reference. Moreover, the mean value of 110 ROC areas at each spatial overlap rate/temporal accuracy level was calculated. For each spatial overlap rate, the 110 ROC areas of TSCICA, TCICA and SCICA were obtained from 11 datasets×10 TSCICA applications with different temporal references, 11 datasets×10 TCICA applications with different temporal references and 11 datasets×10 SCICA applications, respectively. For each temporal accuracy, the 110 ROC areas of TSCICA, TCICA and SCICA were obtained from 11 datasets×10 TSCICA applications with different spatial references, 11 datasets×10 TCICA applications and 11 datasets×10 SCICA applications with different spatial references, respectively. The nonparametric Wilcoxon tests for paired samples were performed to further compare the difference in the ROC area between TSCICA and SCICA/TCICA_at each spatial overlap rate/temporal accuracy.

To investigate the effect of the spatial error rate on TSCICA, SCICA and TCICA, 100 spatial references from SRef2 with varied spatial overlap rates and error rates were used. Moreover, two temporal references from TRef, one with high accuracy (CC = 0.8) and the other with low accuracy (CC = 0.6), were considered. When the temporal reference with high accuracy was selected, TSCICA/TCICA/SCICA was applied to each dataset 100 times to automatically extract the desired task-related component. For each application, one spatial reference from SRef2 was used as a spatial constraint of TSCICA/SCICA, and the temporal reference with high accuracy was used as temporal constraint of TSCICA and TCICA. Because TCICA did not need spatial reference, TCICA was performed 100 times to each dataset using the same temporal reference with high accuracy. The same processing was performed when the temporal reference with low accuracy was selected. The ROC area of the task-related component was recorded. The mean value of 110 ROC areas was calculated at each level with a specific spatial error rate and high/low temporal accuracy. For each spatial error rate in the case of high temporal accuracy, the 110 ROC areas of TSCICA, SCICA and TCICA were obtained from 11 datasets×10 TSCICA/SCICA applications using spatial references with different overlap rates and the temporal reference with high temporal accuracy and 11 datasets×10 TCICA applications using the temporal reference with high temporal accuracy, respectively. For each spatial error rate in the case of low temporal accuracy, the 110 ROC areas of TSCICA, SCICA and TCICA were obtained from 11 datasets×10 TSCICA/SCICA applications using spatial references with different overlap rates and the temporal reference with low temporal accuracy and 11 datasets×10 TCICA applications using the temporal reference with low temporal accuracy, respectively. The nonparametric Wilcoxon tests for paired samples were performed to further compare the difference in the ROC area between TSCICA and SCICA/TCICA at each spatial error rate in the case of high/low temporal accuracy.

#### Determination of the penalty parameters

In this simulation, 11 simulated datasets with different CNR that were generated in the above simulation were used. Because both temporal and spatial constraints were used in TSCICA, two penalty parameters, γ_1_ acting on spatial constraints and γ_2_ acting on temporal constraints, had to be determined. When only one type of constraints is used, the penalty parameter γ can be set to 0.1×4^(k−1)^, as was the case in the previous study [17]. In our study, we set γ_1_ to 0.1×4^(k−1)^ for both TSCICA and SCICA. It is essential for TSCICA to seek an optimal penalty parameter (γ_2_) for the temporal constraint. We set γ_2_ = C×4^(k−1)^ so that γ_1_ and γ_2_ had the same order of magnitude. C is a constant coefficient of γ_2_.

C of the penalty parameter (γ_2_) was varied from 0.1 to 1, with an increment of 0.1. Given that γ_2_ only acts on the temporal constraint, a fixed spatial reference with a medium spatial overlap rate (0.05) was used. For a specific value of C, TSCICA that used different temporal reference from TRef as the temporal constraint and the spatial reference with 0.05 overlap rate as the spatial constraint was applied to each dataset separately to automatically estimate the desired component. The ROC area of each TSCICA processing was recorded. The mean of 110 ROC areas (11 datasets×10 TSCICA applications) was calculated for each C.

#### Comparison between TSCICA and GLM

Although TSCICA depends on both the temporal and spatial prior information, this method still largely differs from GLM. A simple simulated experiment was conducted to demonstrate the differences between TSCICA and GLM. It was assumed that the simulated experiment included one task and that the task induced two task-related spatial components. The experimental paradigm was identical to the above simulated experiments. Two ROIs were predefined in the SimTB toolbox (see Fig. 1C). The ROI1/ROI2 was assumed to be engaged in the first/second task-related component. The simulated fMRI responses, which were added to the two ROIs, were derived from a convolution of the stimulus paradigm with the two different HRFs (see Fig. 1D). Suppose P1 and P2 represent the vector of seven parameters of the two HRFs underlying the time courses that were added to ROI1 and ROI2. We set P1 = [14 8 2 2 6 0 32] and P2 = [6 16 1 1 6 0 32]. The CNR of the simulated dataset was set to 0.01.

The TSCICA method was applied to the simulated data. The spatial reference that was added to TSCICA was assumed to include some prior information of ROI1 (see Fig. 1E). The temporal reference was derived from a convolution of the stimulus paradigm with a HRF that is different from the HRF added to ROI1/ROI2 (see Fig. 1F). The vector of seven parameters (P) of the HRF underlying the temporal reference were set as P = [8 8 2 2 6 0 32]. The target component was transformed into Z score. The voxels with a Z score that was higher than 2 were considered activated. Moreover, the data were inputted into the SPM8 software for GLM analysis. A one-sample t-test was performed to identify the brain regions that were activated by the task compared with the rest condition. The significance level of the t-test was set to p<0.001 without correction.

### 3. The real fMRI experiment

A real fMRI experiment was performed to further demonstrate the feasibility of the proposed method and to compare the performances of TSCICA, TCICA, SCICA and FastICA.

#### Participants

Thirteen volunteer participants (seven females and six males, mean age 23±2 years) participated in the fMRI experiment. All of the subjects were right-handed and had normal vision. The handedness of each subject was confirmed in focused interviews using the Edinburgh inventory [28].

#### Imaging parameters

Brain scans were performed at the MRI Center at Beijing Normal University using a 3.0-T Siemens whole-body MRI scanner. A single-shot T2*-weighted gradient-echo EPI sequence was used for functional imaging acquisition using the parameters: TR/TE/flip angle = 2000 ms/30 ms/90°, acquisition matrix = 64×64; field of view (FOV) = 240 mm; and slice thickness = 3.6 mm, with an inter-slice gap = 0.6 mm. Thirty-three axial slices that were parallel to the AC-PC line were obtained in an interleaved order to cover the entire cerebrum and cerebellum.

#### Experiment design

The entire session lasted 270-s and consisted of five 30-s rest blocks, which were alternated with four 30-s task blocks. During the rest blocks, the subjects were required to relax with their eyes open. During the task blocks, the subjects were required to tap their thumb with their index finger according to their own rhythm.

#### Preprocessing

The serial functional images of each subject were first realigned [29], spatially normalized [30] into the standard Montreal Neurological Institute (MNI) space and resliced into 3×3×4 mm^3^ voxels. The normalized functional images were then smoothed using an 8×8×8 mm^3^ full width at half-maximum (FWHM) Gaussian kernel [16], [31], [32].

#### Data analysis

**Figure 2:**
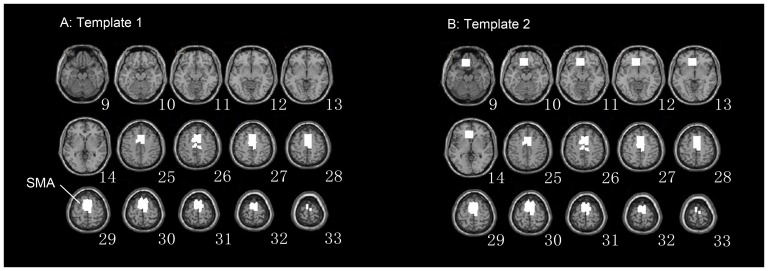
The spatial references that were used in the real fMRI data. (A) The ROIs in the first spatial reference. (B) The ROIs in the second spatial reference.

The temporal reference was derived from the convolution of the design time course with the default HRF. Two spatial references were constructed to further verify the impact of the spatial reference signal on the results that were estimated by TSCICA and SCICA. A tool named WFU_pickAtlas [33], [34], which allows the user to create masks by selecting different areas of the brain, was used to generate the spatial references in our study. The first spatial reference included the supplementary motor area (SMA) as the ROI (see Fig. 2A). The second spatial reference included both the SMA and the artifact ROI, which was defined as a 27×27×24 mm cuboid and centered on the MNI coordinates [−10, 33, −9] (see Fig. 2B).

After preprocessing, TSCICA, TCICA, SCICA, and FastICA were performed separately on the fMRI data from each subject. All of the parameters for the four algorithms were identical to those parameters that were used in the simulation. To automatically extract the desired task-related component, the first/second spatial reference was used as the spatial constraint for TSCICA and SCICA. The temporal reference was used as the temporal constraint for TSCICA and TCICA. Using the same criteria as the simulation, the fMRI data of each subject were reduced by PCA, with 99.9% of the total variance retained before the TSCICA, TCICA, SCICA and FastICA processing. For FastICA, the number of components varied from 26 to 36 across subjects after PCA reduction. The task-related component estimated by FastICA was selected based on the same criteria that were used in the simulation. The subsequent group analysis of the task-related component was conducted using the one-sample t-test program in the software SPM8 to identify the brain regions that were engaged in the task-related component.

Additionally, the GLM analysis was applied to each dataset. The data were processed by a high-frequency filter and by global scaling using the software SPM8. The group data were analyzed using a random effects model. A one-sample t-test was performed to identify the brain regions that were activated by the task compared with the rest condition. All of the statistical results from the four methods were corrected for multiple comparisons via a false discovery rate (FDR) at p<0.01 [35].

Furthermore, a quantitative evaluation of the compactness of the clusters of independent component estimation was used in this study to compare the stability of TSCICA, SCICA, TCICA and FastICA. The ICA estimation were repeated 20 times for each subject and analyzed using the ICASSO software package [36] to evaluate the cluster quality index. The cluster quality index reflects the compactness and isolation of a cluster. It is computed as the difference between the average intra-cluster similarities and average extra-cluster similarities. A cluster quality index close to 1 indicates that the result is consistent and stable.

## Results

### 1. Simulation

#### Robustness to noise magnitude

In this simulation, there were ten simulated datasets for each CNR level. Moreover, Two temporal references with high accuracy (CC = 0.8) and low accuracy (CC = 0.6) from TRef and two spatial references with high spatial overlap rate (8%) and low overlap rate (3%) from SRef1 were considered.

**Figure 3:**
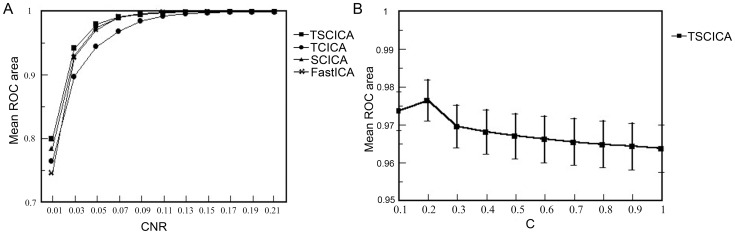
Results of the robustness to noises and determining the penalty parameters. (A) The variation of the mean ROC area with CNR for TSCICA, TCICA, SCICA and FastICA. (B) The variation of the mean ROC area with C of the temporal penalty parameter γ2. The error bar represents the standard error of the mean.

The results of the robustness to noise are shown in Fig. 3A. It can be seen that all methods had high spatial detection power at the low noise levels. Moreover, TSCICA exhibited the best detection power among the four methods at the high noise levels. The results of the nonparametric Wilcoxon tests for paired samples are listed in Table 1. For the CNR that was less than 0.05, the detection power of TSCICA was significantly higher than SCICA, TCICA and FastICA. Moreover, TSCICA showed significantly higher detection power than TCICA when the CNR was lower than 0.13.

**Table 1:** Results of the nonparametric Wilcoxon tests for paired samples in CNR condition.

	Z_(TSCICA-TCICA/SCICA/FastICA)_		
CNR	TCICA	SCICA	FastICA
0.01	5.094*	3.576*	8.185*
0.03	5.423*	2.830*	2.783*
0.05	4.760*	1.925	2.403*
0.07	3.828*	0.903	0.687
0.09	3.283*	0.498	−0.452
0.11	2.648*	0.494	−0.529
0.13	1.537	0.353	−0.308
0.15	0.938	−0.210	0.254
0.17	0.663	0.118	0.184
0.19	0.528	0.068	−0.028
0.21	0.435	−0.107	0.082

Note:asterisk represents P<0.01.

Moreover, the spatial activation of the target component and the corresponding time course extracted from one simulated dataset (CNR = 0.03) by the four ICA methods are shown in the Fig. S1. It can be seen that the activated map of the target component estimated by the four ICA methods mainly located in the predefined ROI. Moreover, the corresponding time courses are highly correlated with the predefined temporal reference. Among the four ICA methods, the ROC area of the target component that was estimated by TSCICA was the highest. Thus, TSCICA produced much less false activation than TCICA, SCICA and FastICA.

#### Robustness to spatial overlap rate, temporal accuracy and spatial error rate

This simulation included 11 simulated datasets with different CNRs that varied from 0.01 to 0.21, with an increment of 0.02. For robustness to the spatial overlap rate/temporal accuracy, ten temporal references from TRef and ten spatial references from SRef1 were considered. For robustness to the spatial error rate, 100 spatial references from SRef2 with varied spatial overlap rates and error rates were used. Moreover, two temporal references from TRef, one with high accuracy (CC = 0.8) and the other with low accuracy (CC = 0.6), were considered.

**Figure 4:**
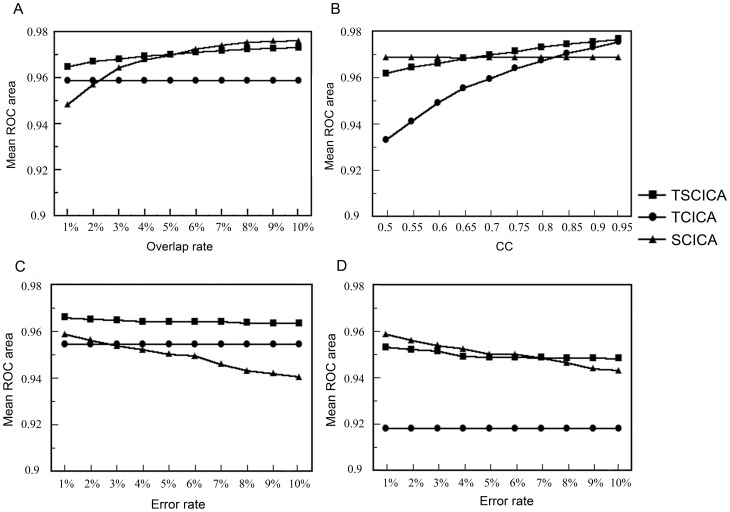
Results of the robustness to spatial overlap rate, temporal accuracy and spatial error rate. (A) The variation of the mean ROC area with overlap rates for TSCICA, TCICA and SCICA. (B) The variation of the mean ROC area with temporal reference for TSCICA, TCICA and SCICA. CC represents the correlation coefficient between the temporal reference and the true time course. (C) The variation of the mean ROC area with error rate in the case of a high temporal accuracy (CC = 0.8) for TSCICA, TCICA and SCICA. (D) The variation of the mean ROC area with error rate in the case of a low temporal correlation(CC = 0.6) for TSCICA, TCICA and SCICA. CC represents the correlation coefficient between the temporal reference and the true time course.

Fig. 4A shows the results of robustness to the spatial overlap rate. The detection powers of TSCICA and SCICA were increased with the increasing spatial overlap rate. Because the TCICA method only introduced temporal constraints on the mixing matrix, the detection power of TCICA did not vary with the spatial overlap rate. Compared with TCICA, TSCICA exhibited greater spatial detection power for all the spatial overlap rates, and SCICA showed larger detection power in the case of the spatial overlap rate that was larger than 2%. Moreover, the spatial detection power of TSCICA was much larger than SCICA for the low overlap rate and slightly lower for the high overlap rate. Table 2 shows results of the nonparametric Wilcoxon tests for paired samples. It can be seen that TSCICA displayed significantly greater detection power than TCICA for all the spatial overlap rates. Moreover, the detection power of TSCICA was significantly larger than that of SCICA for the spatial overlap rate that was less than 0.03 and significantly smaller than that of SCICA for the overlap rate that was larger than 0.07.

**Table 2:** Results of the nonparametric Wilcoxon tests for paired samples in overlaprate condition.

	Z_(TSCICA-TCICA/SCICA)_	
Overlaprate	TCICA	SCICA
0.01	8.628*	9.538*
0.02	8.694*	8.763*
0.03	8.726*	3.082*
0.04	8.804*	0.928
0.05	8.926*	0.030
0.06	9.006*	−1.059
0.07	9.129*	−2.380*
0.08	9.196*	−2.635*
0.09	9.334*	−2.903*
0.10	9.404*	−2.875*

Note:asterisk represents P<0.01.

The variation of the mean ROC area with the accuracy of the temporal reference is displayed in Fig. 4B. Due to the lack of the temporal constraint for SCICA, the accuracy of the temporal reference did not have an impact on the detection power of SCICA. However, the detection power of TCICA and TSCICA increased with the increasing accuracy of the temporal reference. The spatial detection power of TSCICA was invariably larger than that of TCICA. Compared with SCICA, the spatial detection power of TSCICA was larger for the temporal reference with relatively high accuracy (CC>0.65) and lower for the temporal reference with low accuracy. Moreover, SCICA showed much better detection power than TCICA in most cases, except when the temporal reference had extremely high correlation (CC>0.8) with the true time course. The results of the nonparametric Wilcoxon tests for paired samples are listed in Table 3. TSCICA manifested significantly greater detection power than TCICA for most temporal accuracies, except in the case of extremely high temporal accuracy (CC = 0.95). In contrast to SCICA, TSCICA showed significantly larger detection power for high temporal accuracy (CC>0.7) and significantly smaller detection power for low temporal accuracy (CC<0.65).

**Table 3:** Results of the nonparametric Wilcoxon tests for paired samples in CC condition.

	Z_(TSCICA-TCICA/SCICA)_	
CC	TCICA	SCICA
0.5	8.937*	−4.329*
0.55	8.329*	−3.876*
0.6	7.530*	−2.514*
0.65	6.284*	−0.663
0.7	4.989*	1.264
0.75	4.238*	2.796*
0.8	3.886*	3.584*
0.85	3.539*	3.904*
0.9	2.936*	4.321*
0.95	1.628	4.560*

Note:asterisk represents P<0.01.

Fig. 4C and D display the variation of the mean ROC area with the spatial error rate. In the case of the low/high temporal accuracy, the spatial detection power of TCICA remained fixed and that of TSCICA remained nearly fixed for all of the error rates, whereas the spatial detection power of SCICA decreased rapidly as the error rate increased. In contrast to SCICA, TSCICA exhibited better detection power in most cases, except in the case of an error rate that was less than 0.06 with a low temporal accuracy (see Fig. 4D). Moreover, compared with TCICA, greater spatial detection power was invariably observed for TSCICA. For the temporal reference with high accuracy, TSCICA exhibited better detection power at all the error rate levels, and TCICA exhibited better detection power in the case of the error rate that was larger than 0.02, in contrast to SCICA. For the temporal reference with low accuracy, the detection of TSCICA and SCICA is better than TCICA at all the error rate levels. Moreover, when the error rate was larger than 0.06, TSCICA performed better than SCICA. Table 4 lists the results of the nonparametric Wilcoxon tests for paired samples. In the case of high temporal accuracy (CC = 0.8), the detection power of TSCICA was significantly larger than TCICA and SCICA for all the spatial error rates. In the case of low temporal accuracy (CC = 0.6), the detection power of TSCICA was significantly larger than TCICA for all the spatial error rates and significantly larger than SCICA for high spatial error rates (>0.07). Moreover, SCICA displayed significantly higher detection power than TSCICA when the spatial error rate was less than 0.05.

**Table 4:** Results of the nonparametric Wilcoxon tests for paired samples in errorrate condition.

r = 0.8	Z_(TSCICA- SCICA)_		r = 0.6	Z_(TSCICA- SCICA)_	
Errorrate	TCICA	SCICA	Errorrate	TCICA	SCICA
0.01	5.423*	3.497*	0.01	8.764*	−3.908*
0.02	5.259*	4.975*	0.02	8.402*	−3.175*
0.03	5.030*	5.147*	0.03	8.383*	−2.301*
0.04	4.903*	5.534*	0.04	7.643*	−2.514*
0.05	4.858*	6.028*	0.05	7.569*	−0.935
0.06	4.850*	6.173*	0.06	7.428*	−0.860
0.07	4.694*	6.523*	0.07	7.383*	0.354
0.08	4.521*	6.839*	0.08	7.196*	2.451*
0.09	4.395*	7.010*	0.09	7.185*	3.078*
0.10	4.198*	7.358*	0.10	7.177*	3.564*

Note:asterisk represents P<0.01.

#### Determination of the penalty parameters

This simulated experiment used the same 11 simulated datasets with different CNR levels as the above simulation. A fixed spatial reference with a medium spatial overlap rate (0.05) and ten temporal references from TRef were used.

The variation of the mean ROC area with C is presented in Fig. 3B. The results showed that the mean values of the ROC areas were extremely close. The mean ROC area was the greatest when C was equal to 0.2. Thus, 0.2 was selected as the optimal γ_2_ value in TSCICA/TCICA for both the entire simulation and the real fMRI experiment.

#### Comparison of TSCICA and GLM

**Figure 5:**
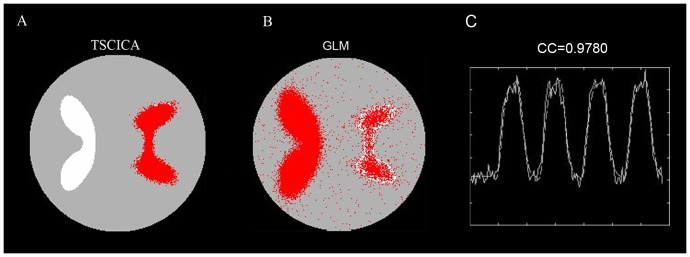
Results of the comparison of TSCICA and GLM. (A) The spatial activation map of the target component that was extracted by TSCICA. (B) The spatial activation that was detected by GLM. (C) The temporal reference (dotted line) and the time course (solid line) that correspond to the target component that was extracted by TSCICA. CC is the correlation coefficient between the estimated time course and the temporal reference.

One simulated dataset that contained two spatial components related to one task were utilized in this simulated experiment. Fig. 5 shows the results of the TSCICA and GLM methods. Because the spatial template contained part of the regions in ROI1, the first task-related component, whose activation map in ROI1, was separated from the data by TSCICA. In contrast, the activated regions detected by GLM contained both ROI1 and ROI2 that should belong to the two different networks.

### 2. The real fMRI experiment

**Figure 6:**
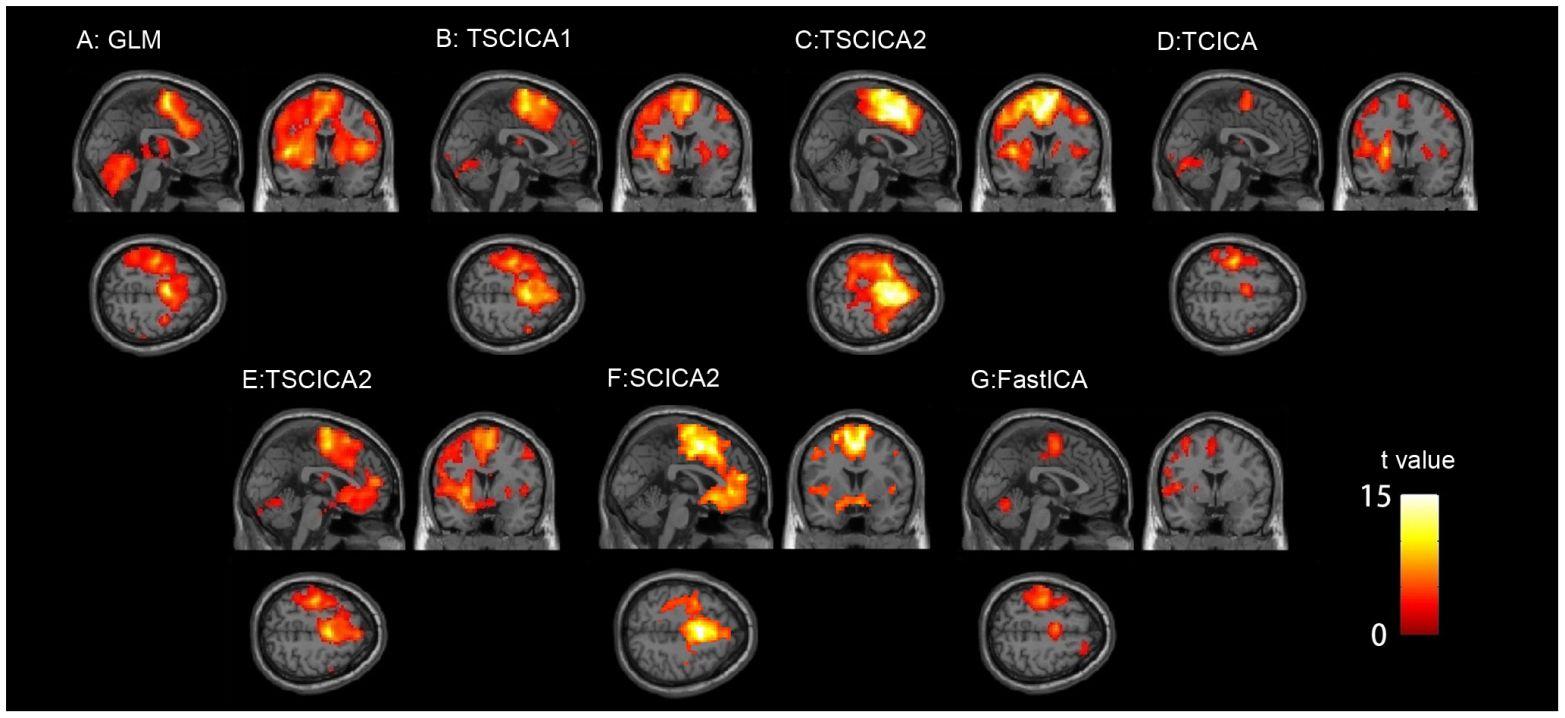
The spatial activation that was estimated by all of the methods. (A) The activated regions that were detected by GLM. (B) The activated regions of the target component that were estimated by TSCICA using the first spatial reference. (C) The activated regions of the target component that were estimated by SCICA using the first spatial reference. (D) The activated regions of the target component that were estimated by TCICA. (E) The activated regions of the target component that were estimated by TSCICA using the second spatial reference. (F) The activated regions of the target component that were estimated by SCICA using the second spatial reference. (G) The activated regions of the target component that were estimated by FastICA.

Fig. 6A shows the result of the GLM method. The brain regions that were associated with motor execution, including the contralateral primary motor cortex (M1), the contralateral premotor areas (PMA), the bilateral supplementary motor areas (SMA), and the cerebellum, were significantly activated by the finger tapping task. The results that were estimated by TSCICA, TCICA, SCICA and FastICA are presented in Fig. 6B–G. TSCICA1/SCICA1 represents the TSCICA/SCICA method using the first spatial reference and TSCICA2/SCICA2 represents the TSCICA/SCICA method using the second spatial reference. The activated regions that were detected by TSCICA1, TSCICA2, and SCICA1 largely overlapped with the regions that were estimated by GLM (see Fig. 6B, C and E). Moreover, TCICA and FastICA detected a much smaller activation than GLM. The inclusion of some artifacts in the second spatial reference led to a largely different result from SCICA2 compared with the other methods (see Fig. 6F).

**Figure 7:**
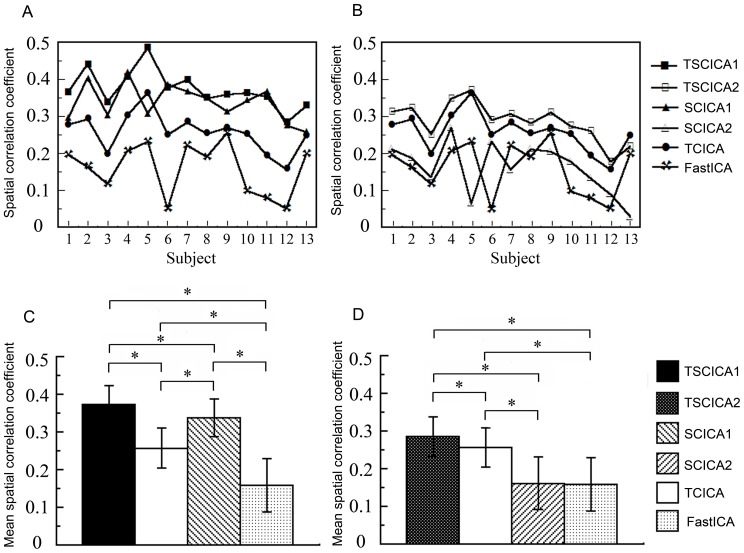
A comparison of TSCICA, TCICA, SCICA and FastICA. (A) The spatial correlation coefficient of TSCICA1, TCICA, SCICA1 and FastICA for an individual subject. (B) The spatial correlation coefficient of TSCICA2, TCICA, SCICA2 and FastICA for an individual subject. (C) The mean spatial correlation coefficients of TSCICA1, TCICA, SCICA1 and FastICA. (D) The mean spatial correlation coefficients of TSCICA2, TCICA, SCICA2 and FastICA. The error bar represents the standard deviation. TSCICA1/SCICA1 represents TSCICA/SCICA using the first spatial reference. TSCICA2/SCICA2 represents TSCICA/SCICA using the second spatial reference. The error bar represents the standard deviation. Note: asterisk represents P<0.01.

The spatial correlation coefficients of the group t-map between GLM and TSCICA1, SCICA1, TCICA, TSCICA2, SCICA2 and FastICA were 0.6226, 0.4960, 0.4762, 0.4847, 0.2486 and 0.1915, respectively. The results from FastICA and SCICA2 exhibited a low correlation with the results from GLM. The spatial correlation coefficient of the Z-score of the task-related component that was estimated by each ICA method and the t-map of the GLM method was calculated for each individual subject (see Fig. 7A and B). It can be seen that the results of TSCICA were more correlated with the group GLM result than the SCICA, TCICA and FastICA results for most subjects, regardless of what spatial reference was applied. Using the spatial template 2 as the spatial constraint, the spatial correlation was slightly reduced for TSCICA and largely reduced for SCICA. Moreover, the means and standard deviations of the spatial correlation coefficients that were obtained from TSCICA, TCICA, SCICA and FastICA are displayed in Fig. 7C and D. To further examine the differences in the spatial correlation coefficients between TSCICA, TCICA, SCICA and FastICA, the nonparametric four paired samples Friedman test [37] for four related samples was performed. For those samples that indicated significant differences between the four methods, the nonparametric Wilcoxon test for paired samples [27] was used to further examine the difference between any two methods. The Friedman test revealed that TSCICA, TCICA, SCICA and FastICA exhibited significant differences for the spatial correlation (p<0.001) using both spatial references. Moreover, the nonparametric Wilcoxon test indicated that the mean spatial correlation coefficient of the target IC that was estimated by TSCICA was significantly larger than that of TCICA, SCICA and FastICA for both of the spatial references (p<0.01). In contrast to SCICA, the mean spatial correlation of TCICA was significantly lower for the first spatial reference (p<0.01) and significantly higher for the second spatial reference (p<0.01).

**Figure 8:**
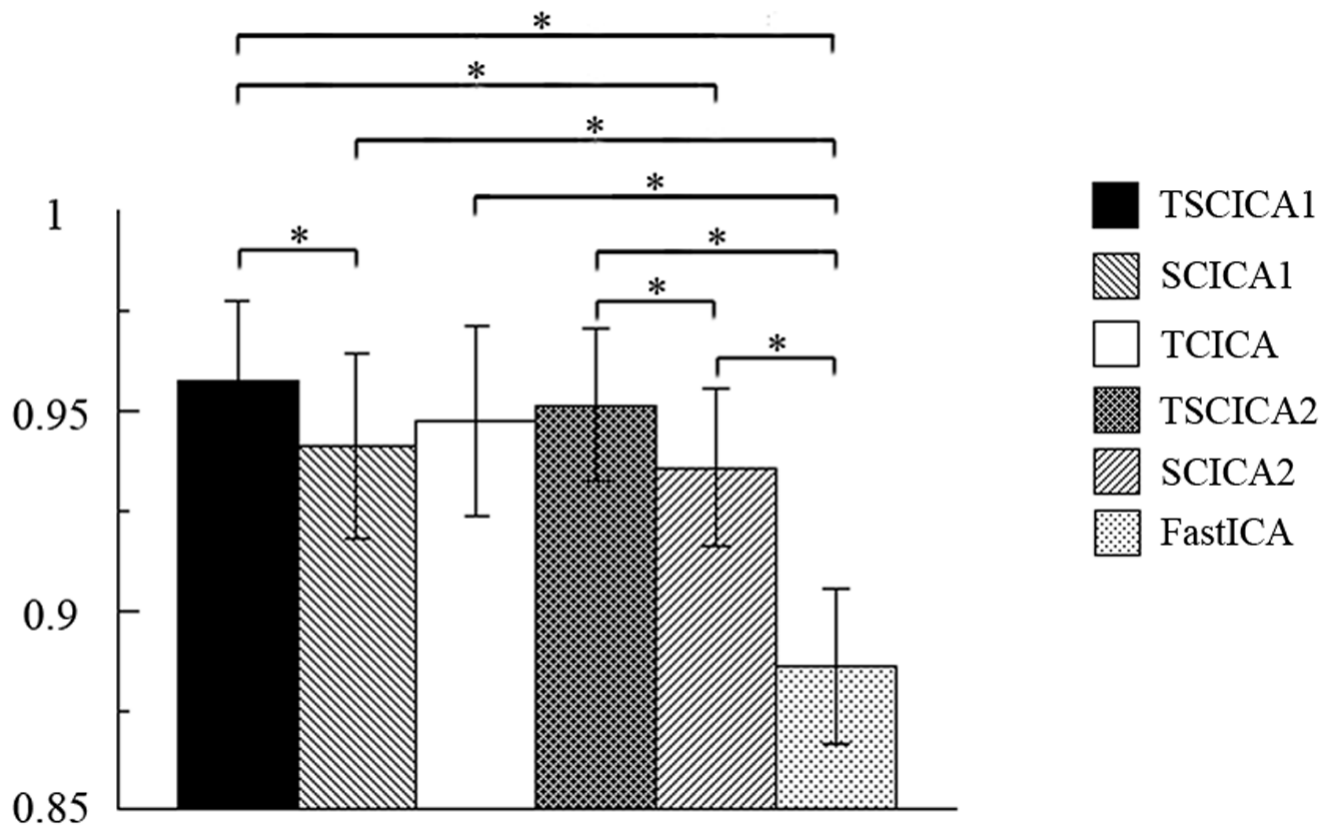
Mean cluster quality index of all the ICA methods. The error bar represents the standard deviation. Note: asterisk represents P<0.01.

Fig. 8 shows the mean of the cluster quality indices across all subjects of each method. To examine the difference of the stability of the target IC estimation among all methods, the nonparametric Wilcoxon test for paired sample was performed. It can be seen that the stabilities of TSCICA, SCICA and TCICA were significantly higher than FastICA (p<0.01). Moreover, the stability of TSCICA was significantly higher than that of SCICA using the first (p<0.01) or second (p<0.01) spatial reference.

## Discussion

In this study, we proposed the TSCICA method by adding prior spatial and temporal information into ICA within the framework of the constrained ICA. We demonstrated the robustness and feasibility of the method under conditions of different noise levels, different temporal references and different spatial references. The results from both simulated and real fMRI data confirm that TSCICA outperformed TCICA, SCICA and FastICA in most cases.

The basic concept of ICA is to extract independent components from data without any prior hypothesis [22]. Estimating all of the independent components from a dataset using the traditional ICA method can result in high computational time costs and the requirement to select the desired components from the entire set of arbitrarily ordered components. In contrast, the CICA method can only extract an interesting subset of ICs if the information available on the ICs can be formulated as reference signals. It has been demonstrated that the CICA method outperforms the classical ICA in computational time, stability and in spatial detection accuracy due to the use of constraints [14]. The CICA method always takes into account only one type of prior information (temporal/spatial) as constraints. However, it is difficult to obtain accurate prior knowledge. Previous research has noted that the shape of the reference signal can have a large influence on the output of an independent component [38]. The proposed TSCICA method used both spatial and temporal information as constraints simultaneously so that one type of prior information moderated the impact of the other type of information on the method. This moderation reduced the dependency of TSCICA on the accuracy of the prior information. Therefore, the inclusion of two types of prior information contributed to the better performance of TSCICA relative to TCICA and SCICA, which only introduce one type of prior information in most cases.

One advantage of ICA over GLM is that this method is powerful for identifying spatially distributed brain networks without any prior hypothesis regarding the data [7]. It has been demonstrated that the brain regions participating in each task, as estimated by GLM, were distributed into multiple functional brain networks relevant to the same task extracted by the ICA method [39]. Our simulated data indicated that the proposed TSCICA method can successfully extract the target brain network engaged in the task from fMRI data, although the task activated two or more brain networks (see Fig. 5). By contrast, GLM identified all regions that were activated by the task, even when the estimated regions responded to different time series that were related to the same task (see Fig. 5). Thus, TSCICA is suitable to the cases in which one or two specific task-related networks need to be examined. It should be noted that the fact that TSCICA depends on prior temporal and spatial information causes the TSCICA method to be not purely exploratory any more. However, TSCICA has two prominent advantages over ICA: (1) automatic extraction of the desired component in a predefined order and (2) a significant decrease in the computational load [14]. Moreover, the results of this study suggest that incorporating both prior temporal and spatial information can largely enhance the performance of ICA. Therefore, TSCICA is attractive to fMRI application, although the exploratory ability of TSCICA was reduced.

Under the condition of high noise levels, the mean ROC area that was produced by TSCICA was larger than those mean ROC areas that were produced by TCICA, SCICA and FastICA, although the mean ROC area showed slight differences among TSCICA, SCICA, TCICA and FastICA for low noise levels (see Fig. 3A). These results suggest that, among the four methods, TSCICA had the best robustness to noise. Furthermore, the variation in the temporal accuracy, the spatial overlap rate and the error rate of the spatial references produced minor impacts on the performance of TSCICA. By contrast, the detection power of TCICA decreased rapidly with the decreasing correlation between the temporal reference and the true time course (see Fig. 4B). The TSCICA method invariably demonstrated better performance than the TCICA method. Moreover, the detection power of SCICA decreased rapidly with the decreasing spatial overlap rate or the increasing error rate (see Fig. 4A, C and D). Compared with the SCICA method, the TSCICA method displayed a better performance in most cases, except for a low temporal accuracy with zero/low error rate and a high spatial overlap rate with zero/low error rate. In contrast to TCICA/SCICA, the better robustness to the temporal/spatial reference that was displayed by TSCICA should be attributed to the additional spatial/temporal constraints that were included in the method. For the real fMRI data, the spatial map of the task-related component that was detected by TSCICA1 and SCICA1 largely overlap. However, the activation pattern of TSCICA1 showed a higher correlation with that of GLM compared with the activation pattern that was produced by SCICA1. When using the second spatial reference that consisted of the regions irrelevant to the task, the spatial activation of SCICA changed greatly, whereas TSCICA performed stably. Compared to TSCICA, TCICA and FastICA detected a much smaller activation and showed a lower correlation with that of GLM. Moreover, the stability of TSCICA was higher than that of SCICA, TCICA and FastICA, regardless of the spatial reference that was applied. Therefore, the results from the real fMRI experiment further verified that TSCICA outperformed TCICA, SCICA and FastICA.

Of note, the convergence of the algorithm depends on the threshold ξ that was used to determine the closeness between the desired IC and the prior information. Lu (2005) noted that the CICA algorithm should use a small ξ initially to avoid any local minima and then should gradually increase its value to converge at the global minimum that corresponds to the only desired independent component when the mean square error (MSE) is used as the closeness measure [14]. One previous study set the threshold ξ to different fixed values at different iterative steps [17]. In this study, the threshold ξ was adaptively altered at each iteration step. When using correlation as the closeness measure, the initial ξ_1_ and ξ_2_ were set to 0.9 to ensure that *w* could converge to the reference rapidly and to avoid any local minima. The threshold ξ_1_ and ξ_2_ were kept fixed for the initial three iterations. For the subsequent iterative steps, the threshold ξ_1_ (ξ_2_) was adaptively set to be slightly larger than the correlation between the spatial reference **R_s_** (temporal reference 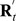) and the signal **Y** (unmixing weights **W**) that was estimated at the previous iterative step. The initial threshold ξ_1_ and ξ_2_ must be changed to a small value if the MSE is used as a closeness measure. Moreover, the threshold ξ_1_ (ξ_2_) was adaptively set to be slightly smaller than the MSE between the spatial reference **R_s_** (temporal reference 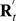) and the signal **Y** (unmixing weights **W**) estimated at the previous iterative step. Compared with the method that set the threshold ξ as a predefined value at each iterative step, the adaptive method that was used in this study can automatically change the threshold according to the correlation/MSE between the estimated signal and the reference.

For the TSCICA method, the two penalty parameters γ_1_ and γ_2_ determined the weights of the spatial and temporal references on TSCICA. If the spatial/temporal penalty parameter were much larger than the temporal/spatial parameter, the spatial/temporal reference would play a predominant role in the algorithm, and the TSCICA would be more similar to SCICA/TCICA. To equalize the impacts of the temporal and spatial references on TSCICA, the two penalty parameters were set to the same order of magnitude. The first penalty parameter, γ_1_, was set to 0.1×4^(k−1)^ based on the pervious study, whereas the second penalty parameter, γ_2_, was determined by the simulated data. In this study, we chose 0.2×4^(k−1)^ as the optimal value of γ_2_ according to the ROC results from the simulation. Good results can be obtained from both the simulated and real fMRI data using the current setting of the two penalty parameters for TSCICA. Moreover, notably, the variation in the mean ROC area is slight when C varied from 0.1 to 1 (see Fig. 3B). Thus, results would be almost stable for C ranging from 0.1 to 1, although 0.2 was selected as the optimal value of C in the study.

The proposed TSCICA method is only suitable for the task fMRI data because prior temporal information is not available from resting fMRI data. In contrast to the prior spatial information, the prior temporal information was much easier to be obtained from the task fMRI data. The temporal reference usually can be generated by convolving the experiment paradigm with the HRF. The spatial reference of the task fMRI data can be generated using some prior information from previous studies. Both the simulated and real experimental demonstrated that a small part of spatial prior information is enough for TSCICA to extract the target component. Moreover, in the real experiment, the spatial reference only includes the prior information of one cluster (SMA). However, the activation map of the target component detected by TSCICA consists of many other clusters including the M1, PMA, and the cerebellum. Thus, the real experiment indicated that TSCICA was able to successfully detect the target component, although there are clusters for which no prior was included in the spatial reference. Because the temporal and spatial references can affect each other, the estimated signal that corresponded to the temporal reference should not be totally independent of the spatial reference. Otherwise, the TSCICA method would not converge. An extreme instance of non-convergence would be that the temporal reference was highly correlated with the time course of one independent component and that the spatial reference was highly correlated with another independent component. The temporal and spatial references that are related to two different independent components separately could easily cause the TSCICA method to fall into an endless loop. In order to avoid the endless loop, the learning rate in TSCICA algorithm was set as 0.98^k^ that was reduced with the increasing of the iterative step. When the iteration step rate is large enough, both the learning rate and the alteration of **W** will be close to zero and the TSCICA algorithm will end the loop. Thus, the algorithm cannot converge to a global optimal value in this case. For the task fMRI data, the temporal reference is derived from the task paradigm, and the spatial reference generally includes the regions that are activated by the task [1]. Therefore, TSCICA easily converges when this method is applied to the task-fMRI data to extract the task-related components. Moreover, the strength of TSCICA is that this method is able to reliably extract the desired component, even if only a very small activated region in the component is included in the spatial reference.

For fMRI task data, the temporal prior information is easier to be obtained than the spatial information. The temporal reference usually can be derived from the convolution of the experimental paradigm with the HRF. However, it is impossible to know the true HRF that underlies the real fMRI data. Due to the departure of the ideal HRF from the true one and to the impact of the various noises on the time courses, the performance of TCICA can be affected to some extent by the inaccuracy of the temporal references. In contrast, the inclusion of some spatial prior information is able to greatly improve the performance and stability of TSCICA.

## Conclusions

In summary, we presented the TSCICA method, which incorporated both the prior spatial and temporal information as constraints on ICA within the CICA framework. The performances of the proposed method, TCICA, SCICA and FastICA were compared using both simulated and real fMRI data. The results indicate that TSCICA was significantly more robust to noise than TCICA, SCICA and FastICA. Moreover, TSCICA displayed better robustness to temporal prior information than TCICA and to spatial prior information than SCICA.

## Supporting Information

Figure S1 **Spatial activation maps and the corresponding time courses for CNR = 0.03.** (A) The activation maps (upper) and the associated time courses (lower) of the target component estimated by TSCICA. (B) The activation maps (upper) and the associated time courses (lower) of the target component estimated by TSCICA. (C) The activation maps (upper) and the associated time courses (lower) of the target component estimated by TSCICA. (D) The activation maps (upper) and the associated time courses (lower) of the target component estimated by TSCICA. The time courses of the target IC were shown in solid line and the time course of the reference was shown in dotted line.
(DOC)

## References

[1] Comon P (1994) Independent component analysis, a new concept? Signal processing 36: 287–314.

[2] Cardoso JF (1998) Blind signal separation: statistical principles. Proceedings of the IEEE 86: 2009–2025.

[3] Calhoun V, Adali T (2004) Semi-blind ICA of fMRI: A method for utilizing hypothesis-derived time courses in a spatial ICA analysis. IEEE 443–452. 10.1016/j.neuroimage.2004.12.012 15784432

[4] Calhoun V, Adali T, McGinty V, Pekar J, Watson T, et al (2001) fMRI activation in a visual-perception task: network of areas detected using the general linear model and independent components analysis. NeuroImage 14: 1080–1088. 11697939 10.1006/nimg.2001.0921

[5] Hyvärinen A, Oja E (2000) Independent component analysis: algorithms and applications. Neural networks 13: 411–430. 10946390 10.1016/s0893-6080(00)00026-5

[6] Stone J, Porrill J, Porter N, Wilkinson I (2002) Spatiotemporal independent component analysis of event-related fMRI data using skewed probability density functions. NeuroImage 15: 407–421. 11798275 10.1006/nimg.2001.0986

[7] McKeown MJ, Makeig S, Brown GG, Jung TP, Kindermann SS, et al (1998) Analysis of fMRI data by blind separation into independent spatial components. Human brain mapping 6: 160–188. 9673671 10.1002/(SICI)1097-0193(1998)6:3<160::AID-HBM5>3.0.CO;2-1 PMC6873377

[8] Bagarinao E, Matsuo K, Nakai T, Sato S (2003) Estimation of general linear model coefficients for real-time application. NeuroImage 19: 422–429. 12814591 10.1016/s1053-8119(03)00081-8

[9] Beckmann CF, DeLuca M, Devlin JT, Smith SM (2005) Investigations into resting-state connectivity using independent component analysis. Philosophical Transactions of the Royal Society B: Biological Sciences 360: 1001–1013. 10.1098/rstb.2005.1634 PMC1854918 16087444

[10] Calhoun VD, Kiehl KA, Pearlson GD (2008) Modulation of temporally coherent brain networks estimated using ICA at rest and during cognitive tasks. Human brain mapping 29: 828–838. 18438867 10.1002/hbm.20581 PMC2649823

[11] Long Z, Chen K, Wu X, Reiman E, Peng D, et al (2009) Improved application of independent component analysis to functional magnetic resonance imaging study via linear projection techniques. Human brain mapping 30: 417–431. 18095282 10.1002/hbm.20515 PMC6870580

[12] Luo J, Hu B, Ling XT, Liu RW (1999) Principal independent component analysis. Neural Networks, IEEE Transactions on 10: 912–917. 10.1109/72.774259 18252587

[13] Lu W, Rajapakse JC (2000) Constrained independent component analysis. In Advances in Neural Information Processing Systems 13 (NIPS2000)

[14] Lu W, Rajapakse JC (2005) Approach and applications of constrained ICA. Neural Networks, IEEE Transactions on 16: 203–212. 10.1109/TNN.2004.836795 15732400

[15] Sun ZL, Shang L (2010) An improved constrained ICA with reference based unmixing matrix initialization. Neurocomputing 73: 1013–1017.

[16] Lin QH, Liu J, Zheng YR, Liang H, Calhoun VD (2010) Semiblind spatial ICA of fMRI using spatial constraints. Human brain mapping 31: 1076–1088. 20017117 10.1002/hbm.20919 PMC2891131

[17] Wang Z (2011) Fixed-point algorithms for constrained ICA and their applications in fMRI data analysis. Magnetic resonance imaging 29: 1288–1303. 21908126 10.1016/j.mri.2011.07.017 PMC3199372

[18] Rasheed T, Lee YK, Kim TS (2009) Constrained Spatiotemporal ICA and Its Application for fMRI Data Analysis. IFMBE Proceedings 23: 555–558.

[19] Formisano E, Esposito F, Kriegeskorte N, Tedeschi G, Di Salle F, et al (2002) Spatial independent component analysis of functional magnetic resonance imaging time-series: characterization of the cortical components. Neurocomputing 49: 241–254.

[20] Bell AJ, Sejnowski TJ (1995) An information-maximization approach to blind separation and blind deconvolution. Neural computation 7: 1129–1159. 7584893 10.1162/neco.1995.7.6.1129

[21] Hyvarinen A (1997) A family of fixed-point algorithms for independent component analysis. Acoustics, Speech, and Signal Processing 1997, ICASSP-97, IEEE International Conference on, IEEE 5: 3917–3920.

[22] Hyvarinen A (1999) Fast and robust fixed-point algorithms for independent component analysis. Neural Networks, IEEE Transactions on 10: 626–634. 10.1109/72.761722 18252563

[23] Bertsekas DP (1982) Constrained optimization and Lagrange multiplier methods. Computer Science and Applied Mathematics. Boston: Academic Press, 1982 1.

[24] Constable RT, Skudlarski P, Gore JC (1995) An ROC approach for evaluating functional brain MR imaging and postprocessing protocols. Magnetic Resonance in Medicine 34: 57–64. 7674899 10.1002/mrm.1910340110

[25] Erhardt EB, Allen EA, Wei Y, Eichele T, Calhoun VD (2012) SimTB, a simulation toolbox for fMRI data under a model of spatiotemporal separability. NeuroImage 59: 4160–4167. 22178299 10.1016/j.neuroimage.2011.11.088 PMC3690331

[26] Gudbjartsson H, Patz S (1995) The Rician distribution of noisy MRI data. Magnetic Resonance in Medicine 34: 910–914. 8598820 10.1002/mrm.1910340618 PMC2254141

[27] Wilcoxon F, Katti S, Wilcox RA (1970) Critical values and probability levels for the Wilcoxon rank sum test and the Wilcoxon signed rank test. Selected tables in mathematical statistics 1: 171–259.

[28] Oldfield RC (1971) The assessment and analysis of handedness: the Edinburgh inventory. Neuropsychologia 9: 97–113. 5146491 10.1016/0028-3932(71)90067-4

[29] Freire L, Mangin JF (2001) Motion correction algorithms may create spurious brain activations in the absence of subject motion. NeuroImage 14: 709–722. 11506543 10.1006/nimg.2001.0869

[30] Friston KJ, Ashburner J, Frith CD, Poline JB, Heather JD, et al (1995) Spatial registration and normalization of images. Human brain mapping 3: 165–189.

[31] Li YO, Adalı T, Calhoun VD (2007) A feature-selective independent component analysis method for functional MRI. International journal of biomedical imaging 2007, 2007; 15635. 10.1155/2007/15635 PMC2233814 18288254

[32] Ma X, Zhang H, Zhao X, Yao L, Long Z (2013) Semi-blind independent component analysis of fMRI based on real-time fMRI system. Neural Systems and Rehabilitation Engineering, IEEE Transactions on 21: 416–426. 10.1109/TNSRE.2012.2184303 22275721

[33] Lancaster J, Summerln J, Rainey L, Freitas C, Fox P (1997) The Talairach Daemon, a database server for Talairach atlas labels. NeuroImage 5: S633.

[34] Lancaster JL, Woldorff MG, Parsons LM, Liotti M, Freitas CS, et al (2000) Automated Talairach atlas labels for functional brain mapping. Human brain mapping 10: 120–131. 10912591 10.1002/1097-0193(200007)10:3<120::AID-HBM30>3.0.CO;2-8 PMC6871915

[35] Genovese CR, Lazar NA, Nichols T (2002) Thresholding of statistical maps in functional neuroimaging using the false discovery rate. NeuroImage 15: 870–878. 11906227 10.1006/nimg.2001.1037

[36] Himberg J, Hyvarinen A (2003) Icasso: software for investigating the reliability of ICA estimates by clustering and visualization. Neural Networks for Signal Processing, 2003. NNSP'03. 2003 IEEE 13th Workshop on, IEEE 259–268.

[37] Friedman BM (1989) Does Monetary Policy Matter? A New Test in the Spirit of Friedman and Schwartz: Comment. NBER Macroeconomics Annual 4: 177–182.

[38] James CJ, Gibson OJ (2003) Temporally constrained ICA: an application to artifact rejection in electromagnetic brain signal analysis. Biomedical Engineering, IEEE Transactions on 50: 1108–1116. 10.1109/TBME.2003.816076 12943278

[39] Long Z, Li R, Wen X, Jin Z, Chen K, et al (2013) Separating 4D multi-task fMRI data of multiple subjects by independent component analysis with projection. Magnetic Resonance Imaging 31: 60–74. 22898701 10.1016/j.mri.2012.06.034

