# Supplementary material for: Temporally and Spatially Constrained ICA of fMRI Data Analysis
Source: PLoS One. 2014 Apr 11;9(4):e94211. doi: 10.1371/journal.pone.0094211 (PMC3984144; doi:10.1371/journal.pone.0094211)
Supplement: Figure S1 — Spatial activation maps and the corresponding time courses for CNR = 0.03. (A) The activation maps (upper) and the associated time courses (lower) of the target component estimated by TSCICA. (B) The activation maps (upper) and the associated time courses (lower) of the target component estimated by TSCICA. (C) The activation maps (upper) and the associated time courses (lower) of the target component estimated by TSCICA. (D) The activation maps (upper) and the associated time courses (lower) of the target component estimated by TSCICA. The time courses of the target IC were shown in solid line and the time course of the reference was shown in dotted line. (DOC) [file pone.0094211.s001.doc]

# Supporting Information


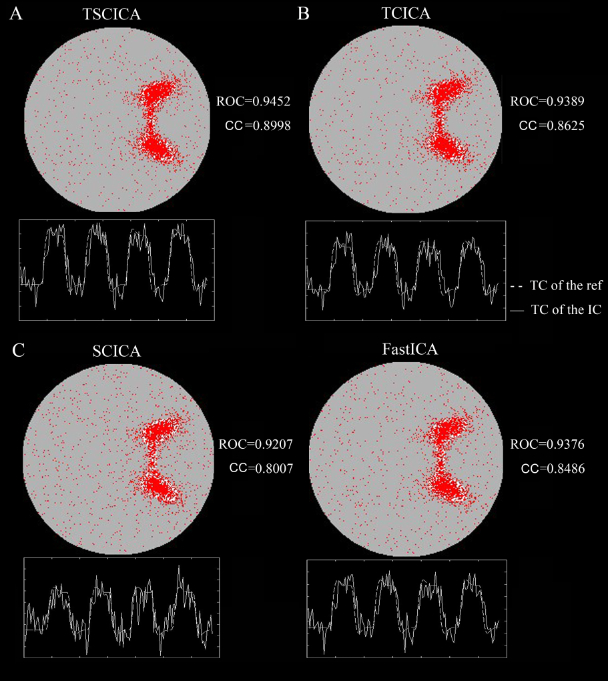


Fig. S1. Spatial activation maps and the corresponding time courses for CNR = 0.03. (A) The activation maps (upper) and the associated time courses (lower) of the target component estimated by TSCICA. (B) The activation maps (upper) and the associated time courses (lower) of the target component estimated by TSCICA. (C) The activation maps (upper) and the associated time courses (lower) of the target component estimated by TSCICA. (D) The activation maps (upper) and the associated time courses (lower) of the target component estimated by TSCICA. The time courses of the target IC were shown in solid line and the time course of the reference was shown in dotted line.
